# Selective pre-priming of HA-specific CD4 T cells restores immunological reactivity to HA on heterosubtypic influenza infection

**DOI:** 10.1371/journal.pone.0176407

**Published:** 2017-05-11

**Authors:** Shabnam Alam, Cory Chan, Xing Qiu, Ian Shannon, Chantelle L. White, Andrea J. Sant, Jennifer L. Nayak

**Affiliations:** 1 Department of Pediatrics, Division of Infectious Diseases, University of Rochester Medical Center, Rochester, New York, United States of America; 2 Department of Biostatistics and Computational Biology, University of Rochester Medical Center, Rochester, New York, United States of America; 3 David H. Smith Center for Vaccine Biology and Immunology, Department of Microbiology and Immunology, University of Rochester Medical Center, Rochester, New York, United States of America; University of Iowa, UNITED STATES

## Abstract

A hallmark of the immune response to influenza is repeated encounters with proteins containing both genetically conserved and variable components. Therefore, the B and T cell repertoire is continually being remodeled, with competition between memory and naïve lymphocytes. Our previous work using a mouse model of secondary heterosubtypic influenza infection has shown that this competition results in a focusing of CD4 T cell response specificity towards internal virion proteins with a selective decrease in CD4 T cell reactivity to the novel HA epitopes. Strikingly, this shift in CD4 T cell specificity was associated with a diminished anti-HA antibody response. Here, we sought to determine whether the loss in HA-specific reactivity that occurs as a consequence of immunological memory could be reversed by selectively priming HA-specific CD4 T cells prior to secondary infection. Using a peptide-based priming strategy, we found that selective expansion of the anti-HA CD4 T cell memory repertoire enhanced HA-specific antibody production upon heterosubtypic infection. These results suggest that the potentially deleterious consequences of repeated exposure to conserved influenza internal virion proteins could be reversed by vaccination strategies that selectively arm the HA-specific CD4 T cell compartment. This could be a potentially useful pre-pandemic vaccination strategy to promote accelerated neutralizing antibody production on challenge with a pandemic influenza strain that contains few conserved HA epitopes.

## Introduction

Influenza is an acute respiratory viral infection that causes annual excess morbidity and mortality in the United States and worldwide [1–6]. This continued high burden of disease despite the availability of an effective vaccine is likely the result of antigenic drift leading to ongoing viral evolution with accumulation of mutations in cell surface viral glycoproteins. Selected changes result in the inability of preexisting neutralizing antibodies to prevent infection, necessitating yearly redesign, manufacture, and administration of vaccine [7]. Additionally, antigenic shift can occur when reassortment between two or more viruses results in the production of a completely novel viral strain that has the potential to cause a worldwide pandemic, such as when a novel swine-origin influenza virus emerged and spread globally in 2009 [8,9]. These ongoing changes repeatedly expose individuals to viral strains that share some, but not all, of their CD4 T cell epitopes with previously circulating viruses.

Following primary influenza infection, a CD4 T cell response of broad specificity develops that includes reactivity to epitopes within all of the major viral proteins [10–13]. On subsequent encounter with an influenza virus that shares some but not all CD4 T cell epitopes with the original infecting strain, memory cells will compete with naïve CD4 T cells specific for novel peptide-epitopes within the virus [14,15]. As memory CD4 T cells are rapidly activated and have less reliance on antigen presentation and costimulatory signaling, they undergo activation early upon viral reexposure [16–20]. Once activated, they can then participate directly in the viral clearance through the secretion of antiviral cytokines that inhibit viral replication and activate the innate immune system, as well as through direct, cell-mediated cytotoxicity [21–27]. These antiviral effector functions contribute to more rapid clearance of virus, destruction of antigen bearing cells, and a shorter duration of antigen presentation [27–31]. As naïve CD4 T cells require a more prolonged period of antigen presentation and generally higher epitope density to be triggered [32–34], this decreased abundance and earlier clearance of antigen could lead to diminished recruitment of novel CD4 T cell specificities.

CD4 T cell help to B cells for the germinal center response depends on peptide display by the antigen specific B cells. A subset of CD4 T cells upregulate CXCR5 and downregulate CCR7, enabling migration to the T-B border and interaction with antigen presenting B cells. If these cells form stable conjugates with their cognate B cell, they can become T follicular helper cells (Tfh) and enter the B cell follicle, where they play a critical role in the initiation and maintenance of the germinal center reaction and the selection of high affinity clones during somatic hypermutation [35–38]. As mutations tend to accumulate within the HA protein as influenza evolves, a failure to recruit novel CD4 T cells is likely to particularly impact cells directed against the HA protein, potentially leading to a loss in HA-specific Tfh, the key CD4 T cell specificity needed for production of high affinity neutralizing antibody [39].

We have previously demonstrated that following secondary infection of X-31 (H3N2) infected mice with x139, a recombinant virus containing the HA, NA, nucleoprotein, and polymerase basic 1 proteins of A/New Caledonia/20/99 (H1N1) with all other proteins derived from the X-31 viral strain, there was a selective loss in CD4 T cell responses directed against novel influenza peptide-epitopes contained predominately within HA protein [14]. This loss in HA-specific CD4 T cell help was associated with a dramatic decline in HA-specific antibody, possibly due to limiting numbers of HA-specific CD4 follicular helper T cells following the secondary infection [35,40–42]. As the production of high affinity, class switched neutralizing antibody is the most commonly accepted correlate of protection from a future infection with an influenza virus of the same strain [43], such a decline in neutralizing antibody post infection could leave individuals susceptible to future viral infections.

In this study, the same model of sequential influenza infection was used to determine the effect of selectively priming CD4 T cell memory against epitopes within the HA protein prior to a heterosubtypic viral challenge. We hypothesized that immunizing with HA peptides prior to the secondary infection would lead to a corresponding increase in HA-specific memory CD4 T cells with the potential to become Tfh. Our results demonstrate that establishing a preexisting pool of memory CD4 T cells against the HA protein led to a partial restoration of the HA-specific antibody response on challenge with a highly divergent influenza viral strain. This suggests that vaccination regimens producing broadly cross-reactive HA-specific memory CD4 T cells have the potential to increase the generation of HA-specific antibodies, potentially leading to the more rapid development of neutralizing antibodies both in situations of limiting antigen as well as on exposure to a pandemic influenza strain against which there is little preexisting B cell memory.

## Materials and methods

### Mice

B10.S-H2^S^/SgMcdJ mice (B10.S; H-2^s^) were purchased from The Jackson Laboratory and bred at the University of Rochester. Animals were housed in specific pathogen-free facilities and maintained according to institutional guidelines. All studies were performed in compliance with the United States Department of Health and Human Services *Guide for the Care and Use of Laboratory Animals*. Studies were approved by the University of Rochester Committee on Animal Resources, Animal Welfare Assurance Number A3291-01. The protocol under which these studies were conducted was originally approved on March 4, 2006 (protocol no. 2006–030) and undergoes reapproval every 36 months. Throughout the course of these experiments, mice were monitored a minimum of twice weekly for ability to ambulate, ability to intake food and water, and signs of discomfort including ruffled fur, hunched posture, and guarding behavior. As a humane endpoint, mice that exhibited signs of undue discomfort were to be euthanized using CO2 asphyxiation followed by secondary cervical dislocation. Generally the influenza infections and immunizations utilized in this study were well tolerated, with no animal requiring euthanasia as a result of experimental procedures.

### Synthetic peptides

Peptides (17-mer) previously identified as I-A^s^–restricted for the HA protein of A/New Caledonia/20/1999 (NR-2602) [12] were obtained through the National Institutes of Health Biodefense and Emerging Infections Research Resources Repository (National Institute of Allergy and Infectious Diseases) or synthesized in our facility using an Apex 396 system (AAPPTec, Louisville, KY) as described previously [44].

### Immunization and influenza infection of mice

X-31(H3N2) influenza virus was provided by Dr. David Topham (University of Rochester) and x139 (H1N1) influenza virus was provided by Dr. Doris Bucher (New York Medical College). Mice 2 to 5 months of age were anesthetized by i.p. injection of Avertin (2,2,2-tribromoethanol) and were infected intranasally with 30 μL of X-31 in PBS at a dose of 300,000 EID_50_ per mouse. After 4–5 weeks, mice were immunized by intraperitoneal injection of a pool of A/New Caledonia/20/99 HA-peptides emulsified in PBS and alum (Alhydrogel, InvivoGen). This peptide pool contained five known HA-derived epitopes, with peptides obtained from BEI Resources (NIAID, NIH; NR-2602) and used at a quantity of 10 nmol each. Peptides used included the following: either HA 21 (120-EQLSSVSSFERFEIFPK-136) or HA 22 (126-SSFERFEIFPKESSWPN-142); HA 25 (144-TVTGVSASCSHNGKSSF-160); HA 28 (162-RNLLWLTGKNGLYPNLS-178); HA 57 (334-LRNIPSIQSRGLFGAIA-350); and HA 66 (386-NAINGITNKVNSVIEKM-402) (Table 1). As a control, mice were immunized with an emulsion of PBS and alum with no peptide added. After 4 more weeks rest, mice were infected intranasally with x139 influenza at a dose of 50,000 EID_50_. Mice were euthanized at various days post infection and spleen and mediastinal lymph nodes were excised and used as a source of CD4 T cells for *in vitro* assays, as described below. Serum was also collected from individual mice by either cardiac puncture or by sub-mandibular bleed to measure antibody responses.

**Table 1:** I-A^s^ peptide-epitope alignment^1^.

I-A^s^ Restricted Nonconserved Epitopes		I-A^s^ Restricted Conserved Epitopes	
HA 120–136:	120-EQLSSVSSFERFEIFPK-136 **SLVA**S**SG---TL**E**FITE**	NP 270–286:	270-VAHKSCLPACVYGPAVA-286 VAHKSCLPACVYGPAVA
HA 126–142:	126-SSFERFEIFPKESSWPN-142 **G---TL**E**FITEGFT**W**TG**	PB1 656–672:	656-EYDAVATTHSWVPKRNR-672 EYDAVATTHSW**I**PKRNR
HA 144–160:	144-TVTGVSASCSHNGKSSF-160 T**QN**G**G**S**NA**C**KRGPG**S**G**F		
HA 162–178:	162-RNLLWLTGKNGLYPNLS-178 **SR**L**N**WLT**KSGST**YP**V**L**N**		
HA 334–350:	334-LRNIPSIQSRGLFGAIA-350 **M**RN**V**P**EK**Q**T**RGLFGAIA		
HA 386–402:	386-NAINGITNKVNSVIEKM-402 **A**AI**DQ**I**NG**K**L**N**R**VIEK**T**		

^1^ Top sequence represents x139; bottom sequence represents x31.

### Elispot assays for cytokine secreting cells

As described previously [10,12], CD4 T cells were analyzed for abundance and specificity using cytokine Elispot assays. 96-well multi-screen HTS filter plates (Millipore, Billerica, MA) were coated with 2μg/mL of purified rat anti-mouse IFNγ (clone AN18, BD Bioscience, San Jose, CA) or 2μg/mL of purified rat anti-mouse IL2 (clone JES6-1A12, BD Bioscience, San Jose, CA) in PBS overnight at 4°C. Individual spleens and mediastinal lymph nodes were collected and processed to a single cell suspension. Splenocytes were depleted of red blood cells by treatment with ACK lysis buffer (0.15M NH_4_Cl, 1mM KHCO_3_, 0.1mM Na_2_EDTA in H_2_O, pH 7.2–7.4) for 5 minutes at room temperature, then were washed and enriched for CD4 T cells by negative selection using MACS CD4 T cell purification (Miltenyi Biotec, Gladbach, Germany) per the manufacturer’s instructions. Syngeneic splenocytes from uninfected mice were used as APCs. CD4 T cells from the spleen or undepleted cells from the MLN were co-cultured with syngeneic splenocytes as APC and recall peptides (Table 1) at a concentration of 10 μM for 16 to 18 hours at 37°C and 5% CO_2_. The plates were processed and analyzed as previously described, with data presented as cytokine Elispots per million CD4 T cells, with background values subtracted.

### Antibody ELISA assays

Mouse sera were collected from individual mice at day 8 post x139 infection and HA-specific antibodies were measured by ELISA using recombinant HA protein derived from A/New Caledonia/20/99 (BEI Resources, NIAID, NIH; NR-42022). Briefly, 96-well polystyrene flat bottom plates (Costar) were coated overnight at 4°C with 300 ng/100 μL HA protein per well. Wells were washed and then were incubated with blocking buffer (3% BSA in PBS) for 1 hour at room temperature. Blocking buffer was removed and serum diluted in 0.5% BSA-PBS was added to the plates and incubated for 2–3 hours at room temperature. The wells were the again washed and developed as previously described [44]. Absorbance at 405nm was read using the SoftMax Pro software on a VMax plate reader. To detect antibodies against the H3 HA protein, the same procedure was used but the plate was coated with the A/Brisbane/10/07 H3 HA protein (BEI Resources, NIAID, NIH; NR-19238).

### Microneutralization assay

Sera collected at 24 days post-terminal infection were treated with receptor-destroying enzyme (Denka Seiken, Tokyo, Japan) per the manufacturer’s protocol and heat-inactivated prior to testing as previously described [44]. Neutralization titers were defined as the reciprocal of the highest serum dilution at which all of the culture wells were negative for cytopathic effect.

### Quantitative PCR

Harvested lung tissue obtained at days 2, 4, and 8 following terminal infection was kept in RNA-later (Ambion) at -20C until processing. RNA was extracted using RNeasy kit (QIAgen) using manufacturers protocols. Extracted RNA concentration was analyzed by spectrometry (NanoDrop 2000, Thermo Scientific) and stored at -80°C until used. RNA samples were thawed to 4°C and cDNA was made using SuperScript IV Reverse-Transcriptase kit (Invitrogen) using manufacturers protocol with 100ng RNA template. qPCR was performed using M1 specific Influenza A primers (BEI Resources, NR-15594, 15595) and probe (BEI Resources NR-15593) with a 6-carboxyfluorescein (6-FAM) reporter and the quencher Black Hole Quencher 1. Positive controls were set up in the same manner using mouse GAPDH primers and probe (Applied Biosystems), and negative controls utilized lung RNA obtained from animals only infected with X-31 at 8 weeks post infection. All samples were run in quadruplicate using a TaqMan PCR master mix (Applied Biosystems), with a standard curve obtained from serially diluting a 1 ng/mL solution of M1 PCR product until the limit of detection was reached. Samples were run in a 96-well qPCR plate (BioRad) on a BioRad CFX96 instrument for 40 cycles using the following protocol: 50°C for 2 minute and 95°C for 10 seconds, followed by 40 cycles at 95°C for 14 seconds and 60°C for 1 minute. qC results were averaged for each sample and plotted along a standard curve to determine the amount, in ng/mL, of viral DNA present in the sample. The molecular weight of the M1 amplicon was determined using NCBI PrimerBlast, allowing for calculation of the number of amplicons per ml.

### Statistical analyses

Two-way group comparisons were evaluated using the Wilcoxon rank-sum test. Multiple group comparisons were performed using the Kruskal-Wallis one-way ANOVA test with the Dunn *post hoc* test, with Holm multiple testing adjustment to control for familywise error rate at an α = 0.05 level. Elisa data were analyzed using a linear mixed effect model for pairwise group comparisons, where *Y*_*ij*_ = Group_*i*,*k*_*β*_*G*,*k*_ + Dilution_*j*_*β*_*D*,*k*_ + *α*_*i*,*k*_ + *ϵ*_*ijk*_. Here *Y*_*ij*_ is the Elisa measurement for the *i*th subject, at dilution level *j*. Group_*i*,*k*_ is a binary variable that indicates the *k*th pairwise group membership of the *i*th subject. Variable Dilution_*j*_ is the dilution level associated with the observation. *α*_*i*,*k*_ is a random effect term that quantifies the within-subject correlation between multiple dilution levels. *ϵ*_*ijk*_ is the *i*.*i*.*d*. measurement error. A regression *t*-test with Satterthwaite approximation of degrees of freedom was used to test the hypotheses *H*_0,*k*_: *β*_*G*,*k*_ = 0, versus *H*_1,*k*_: *β*_*G*,*k*_ ≠ 0. Due to the large numbers of pairwise comparisons, the Benjamini-Hochberg multiple testing adjustment was applied to control false discovery rate (FDR) at 0.05 level. A *p* value of <0.05 was considered statistically significant.

## Results

Given our previous findings of a decrease in HA-specific CD4 T cells and HA-specific antibody following secondary heterosubtypic challenge, we first sought to determine the effect of secondary infection on the availability of viral antigen. We utilized our original model of initially infecting B10.S mice with “X-31” influenza, a recombinant influenza virus containing the hemagglutinin (HA) and neuraminidase (NA) proteins of A/Aichi/2/68 (H3N2), with all other proteins derived from A/Puerto Rico/8/34 (H1N1). After waiting 8 weeks to establish memory, mice were infected with a reassortant virus (“x139”) composed of the HA, NA, nuclear protein (NP) and polymerase basic 1 (PB1) proteins of A/New Caledonia/20/99 virus (H1N1) with all other proteins derived from X-31. This combination of viruses thus express unrelated HA and NA proteins while most internal viral proteins remain conserved. Mice solely infected with X-31 eight weeks prior served as a control for waning CD4 T cell immunity. Lungs were harvested at 2, 4, and 8 days post x139 infection for determination of viral load by quantitative PCR using primers and probe derived from the M1 protein. At 8 days post infection, CD4 T cell responses were also directly compared between secondary and primary x139 infections using an IL-2 Elispot assay and HA-specific antibody levels were assessed using an HA protein ELISA.

**Fig 1:**
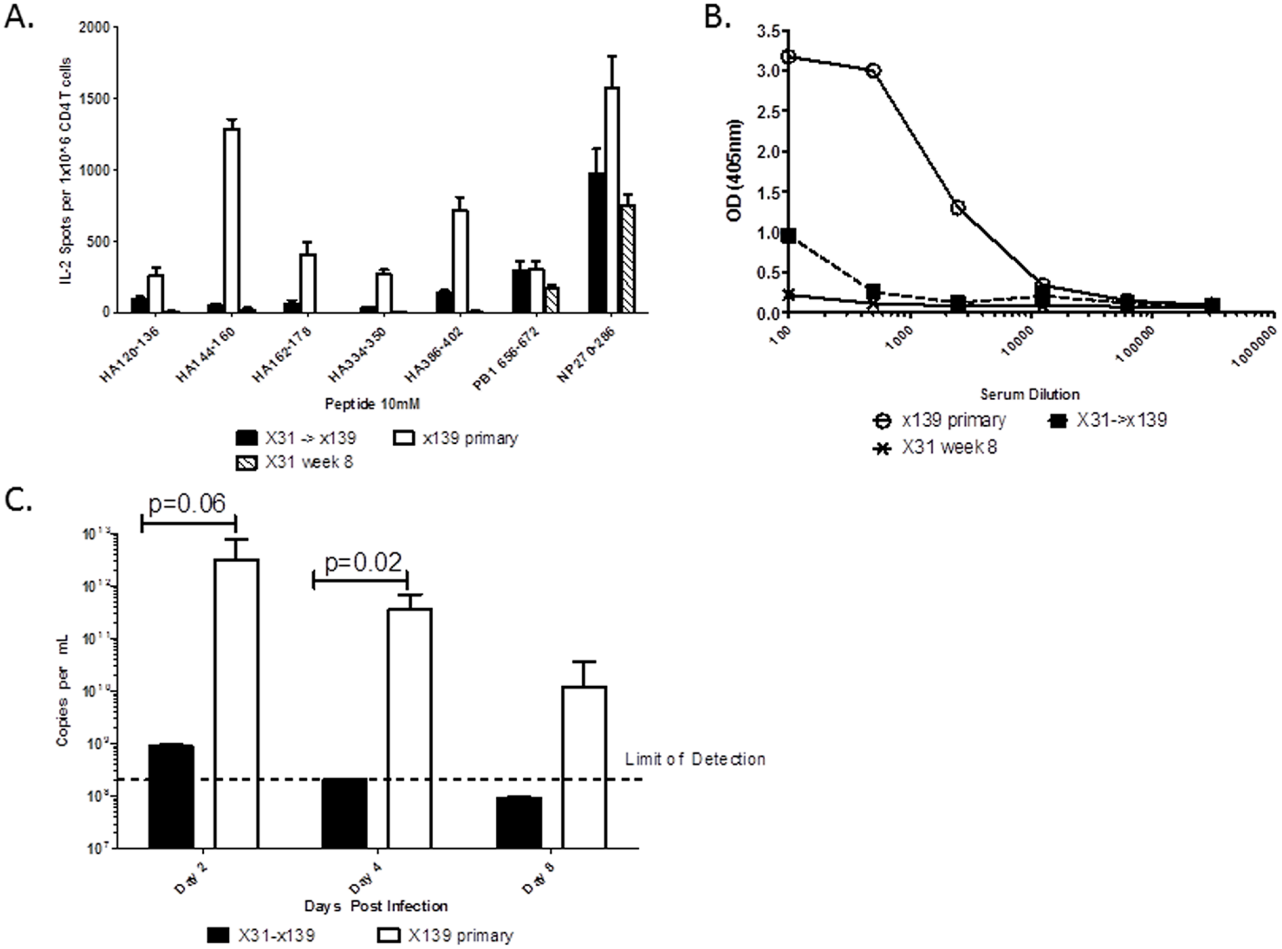
The suppression of novel responses demonstrated following a secondary influenza infection is associated with a marked decline in antigenic load. **A:** Mice were infected with 300,000 EID_50_ of X-31 (H3N2) influenza and then were rested for 8 weeks. These mice, together with a cohort of naïve mice, were then infected with 50,000 EID_50_ of x139 (H1N1) influenza. Mice only infected with X-31 8 weeks prior served as a control for waning immunity. CD4 T cell responses in splenocytes derived from 3 to 4 individual mice per group were quantified at day 8 post x139 infection by IL-2 Elispot assay, with data presented as the average spot count per million CD4 T cells after subtracting background. Error bars represent the standard error of the mean. **B:** Sera from these same 3 to 4 individual mice per group were pooled and the titer of HA-specific antibodies in each group was quantified using an HA ELISA assay, with data presented as the average of duplicate wells. **C:** Viral load in the lungs of 3–4 individual mice per group was determined by quantitative PCR using primers and probe derived from the M1 protein at days 2, 4, and 8 following x139 infection. Samples were run in quadruplicate, with the average qC for each sample determined and plotted on a standard curve. Data are presented as the average number of copies per mL, with error bars representing the standard error of the mean.

As previously described, CD4 T cell responses directed against novel epitopes within the HA protein were greatly diminished in the spleen at 8 days following secondary infection compared to the responses following a primary infection (Fig 1A). This decrease was associated with a substantial decline in the HA-specific antibody response, as measured by ELISA assay (Fig 1B) [14]. We postulated that the failure of naïve CD4 T cells and B cells to initiate responses against new specificities could partially be the result of a decline in antigen load following the secondary infection. To test this hypothesis, viral RNA was measured at days 2, 4, and 8 post-infection and a remarkable decline in detectable M1 nucleic acid was noted after secondary compared to primary x139 infection (Fig 1C). Although a small amount of viral RNA was detected at day 2 post-secondary infection, this rapidly declined and was essentially undetectable by day 4. In contrast, after primary infection viral RNA was still detectable at 8 days post infection. Collectively, these results suggest that diminished viral antigenic abundance following secondary infection is responsible for the selective boosting of memory cells and corresponding loss in recruitment of new CD4 T cell specificities, as the threshold of antigen required for activation of memory CD4 T cells is substantially lower than that needed for activation of naïve CD4 T cells [16–20,32–34].

We next wanted to evaluate the effect that establishing HA-specific memory CD4 T cells prior to secondary challenge would have on the development of CD4 T cell and antibody responses. To complete these experiments, it was essential to selectively establish CD4 T cell memory, as priming and expansion of the HA-specific B cell repertoire prior to secondary challenge would result in formation of B cell memory as well as circulating antibody to HA that could provide sterilizing immunity on secondary challenge. Therefore, we choose to immunize with established CD4 T cell peptide-epitopes, unstructured ligands that would expand the HA-specific CD4 T cell repertoire without simultaneously priming HA-specific B cells [39].

**Fig 2:**
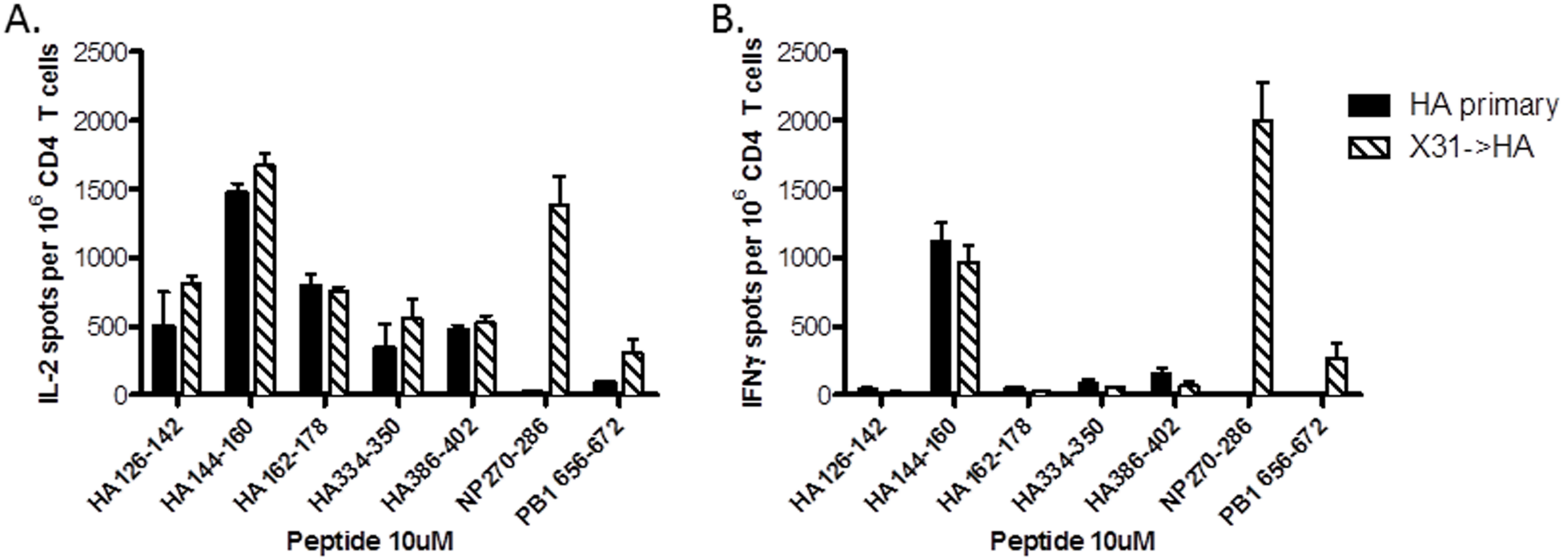
Reactivity following HA peptide priming is not influenced by previous X-31 infection. A cohort of B10.S mice was infected with 300,000 EID_50_ of X-31 (H3N2) influenza and rested for 4 weeks. These previously infected mice, together with a cohort of naïve mice, were then immunized intraperitoneally with a pool of 5 H1 HA peptides in alum as described. Spleens were harvested at day 10 post immunization and CD4 T cells were isolated by MACS cell purification, with reactivity to a panel of selected epitopes determined by Elispot assay. **A:** Reactivity to selected peptide-epitopes as measured by IL-2 Elispot assay; **B:** Reactivity to peptide-epitopes quantified by IFNγ Elispot. Data represent the results obtained from 3–6 individual mice per experiment group, with the spot count normalized to 10^6^ CD4 T cells after subtracting background and then averaged.

We initially evaluated the CD4 T cell responses following peptide immunization alone or in the setting of a prior viral infection to determine if prior viral infection resulted in alterations in the CD4 T cell response magnitude or specificity post-immunization. Naïve mice or mice previously infected with X-31 influenza virus were intraperitoneally administered a pool of 5 known I-A^s^ restricted H1 HA peptide-epitopes in alum (Table 1) [12]. As expected, only the mice previously infected with the X-31 virus had reactivity to epitopes within the conserved internal viral proteins in the spleen, including NP 270–286 and PB1 656–672. Previously uninfected mice generated a robust CD4 T cell response to all of administered HA peptides when cytokine producing cells were quantified with an IL-2 Elispot assay, with the greatest response to the HA 144–160 peptide (Fig 2A). This reactivity was largely unchanged in mice with preexisting memory to the X-31 virus, indicating that prior infection with an H3N2 influenza virus had minimal effect on the response to H1 HA peptide immunization. IFNγ-producing cells were only detected following restimulation with the HA 144–160 peptide and, to a lesser extent, the HA 386–402 peptide regardless of whether memory to X-31 had been established (Fig 2B). Thus, IL-2 Elispots were used to assay CD4 T cell reactivity in all subsequent experiments as this was viewed to be the more comprehensive read-out of CD4 T cell abundance. Through these experiments, it was determined that circulating H1-specific CD4 T cell memory could be established using peptide vaccination, and that the responses to this immunization were unaffected by previous infection with an H3N2 influenza virus.

**Fig 3:**
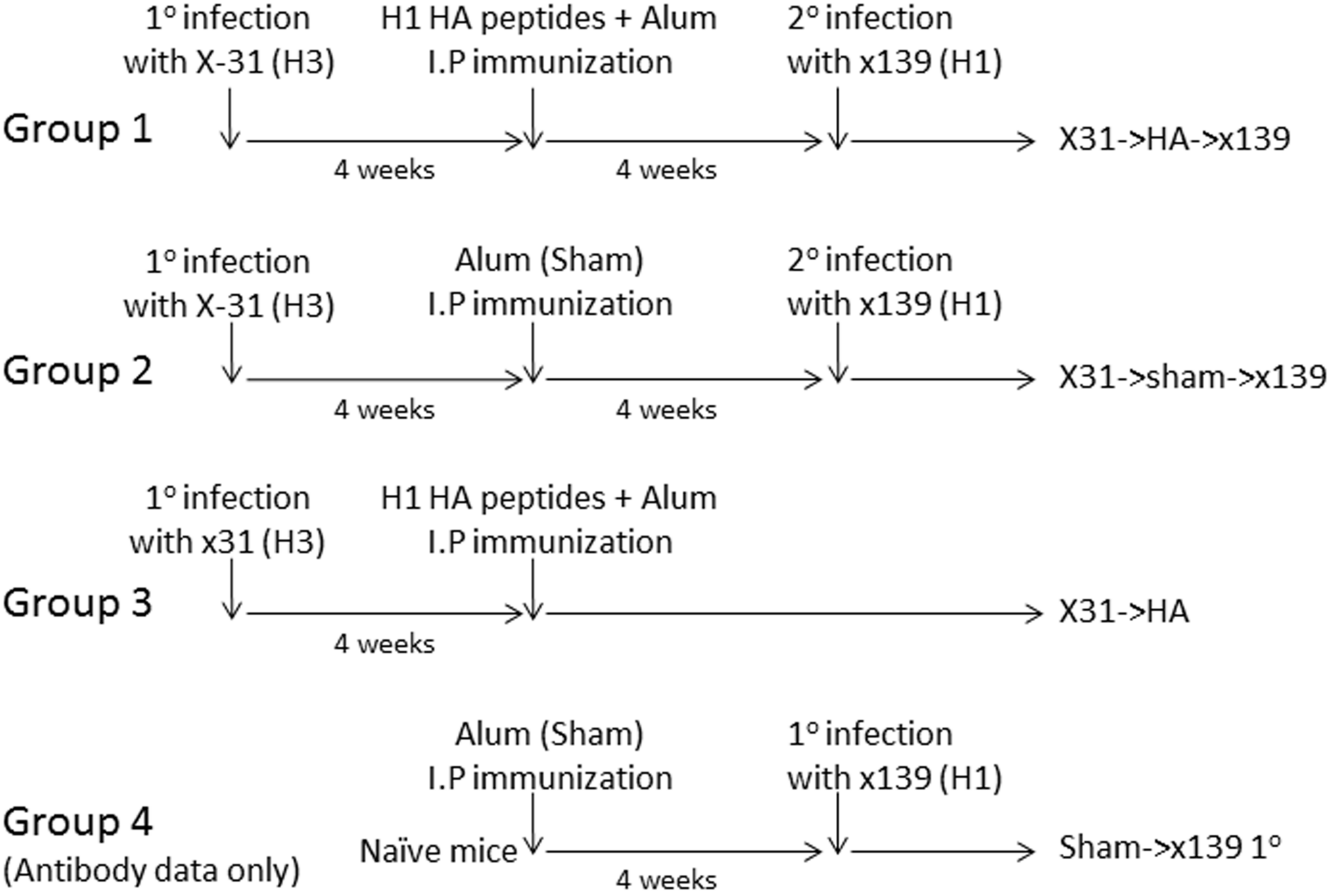
Experimental design to examine the effect of establishing HA-specific memory by peptide immunization prior to secondary infection. B10.S mice were infected with 300,000 EID_50_ of X-31 (H3N2) influenza virus and were rested for four weeks. Groups 1 and 3 were then immunized with a pool of 5 HA peptides in alum IP as described while Group 2 and 4 were only immunized with alum in PBS (sham). After 4 weeks, Groups 1, 2 and 4 were infected with 50,000 EID_50_ of x139 (H1N1) influenza virus. Spleen, mediastinal lymph nodes, and serum were harvested at days 8 or 24 following secondary infection (Group 4 only had serum harvested).

Using this peptide vaccination regimen, we next sought to determine the effect of establishing circulating HA-specific CD4 T cell memory on the CD4 T cell response following a secondary viral challenge. To perform these experiments, a cohort of mice was infected with X-31 (H3N2) influenza. Mice were then rested for 4 weeks, after which a pool of H1 HA peptides was administered IP in alum (Table 1), with sham vaccination used as a control. Four weeks later, two of the previously infected cohorts of mice were infected with the x139 H1N1 virus, while one was left uninfected. Thus, 3 experimental groups were established: HA preprimed mice undergoing secondary infection (Group 1: X31-HA-x139), sham immunized mice undergoing secondary infection (Group 2: X31-sham-x139), and X31 infected mice immunized with the H1 peptide pool but not infected with x139 to control for waning memory (Group 3: X31-HA). Serum was obtained from a 4^th^ group (Group 4: Sham-x139) to determine the antibody levels obtained following a primary infection. This experimental design (Fig 3) allowed a comparison of CD4 T cell reactivity following secondary infection in mice with or without prior H1-specific CD4 T cell memory established.

**Fig 4:**
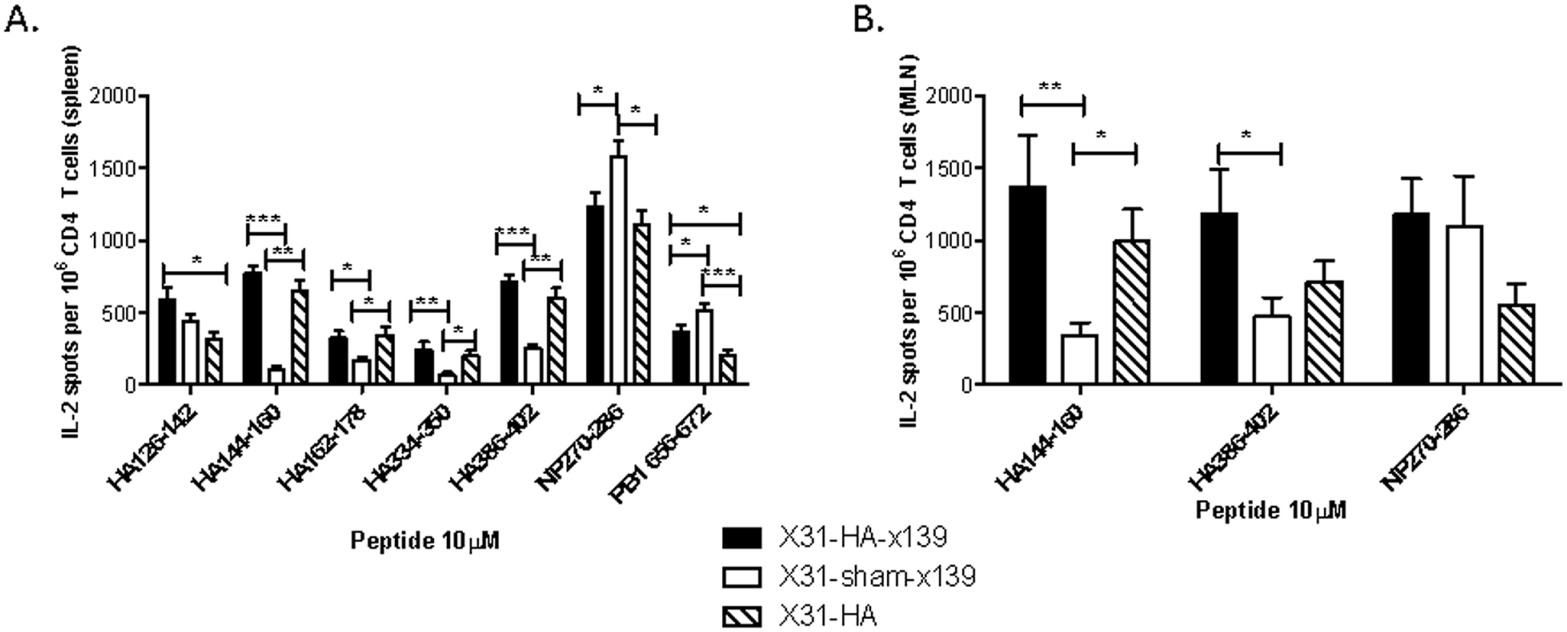
Immunization with a pool of HA peptides restores CD4 T cell reactivity to the selected HA epitopes following a secondary infection. Mice were infected and immunized as depicted in Fig 3. At day 8 following the secondary infection, individual spleens and mediastinal lymph nodes were harvested. CD4 T cells were isolated by MACS cell purification and reactivity to a panel of peptide-epitopes was measured by IL-2 Elispot assay. **A:** CD4 T cell reactivity in the spleen. Data represent results from 9–13 individual mice averaged in each experimental group. **B:** CD4 T cell reactivity within the mediastinal lymph node. Data presented represent the average results from 6–13 individual mice in each experimental group. All data are presented as the spot count normalized to 10^6^ CD4 T cells after subtracting background, with error bars depicting the standard error of the mean. * = p<0.05; ** = p<0.01; *** = p<0.001 by Kruskal-Wallis one-way ANOVA test.

These experiments revealed that mice immunized with HA peptides but not infected with the H1N1 virus (X31-HA cohort) had persisting CD4 T cell memory to peptides included in the immunizing pool at 4 weeks post immunization. These mice also continued to have a readily detectable response to the NP 270–286 and PB1 656–672 epitopes primed by the initial X-31 infection, producing on average 1106 and 204 IL2 spots per 10^6^ spleen-derived CD4 T cells, respectively (Fig 4A and 4B, hatched bars). Only modest reactivity to H1 HA epitopes was seen in the mice that received the sham immunization, with one exception being CD4 T cell reactivity to the HA 126–142 peptide within the spleen (Fig 4, open bars). Notably, mice that had immunological memory to H1 HA epitopes established did not display any suppression of HA reactivity in the spleen or MLN following a secondary viral challenge, with responses following HA prepriming about 3.4 times greater than responses following sham immunization on average (Fig 4, black compared to open bars).

**Fig 5:**
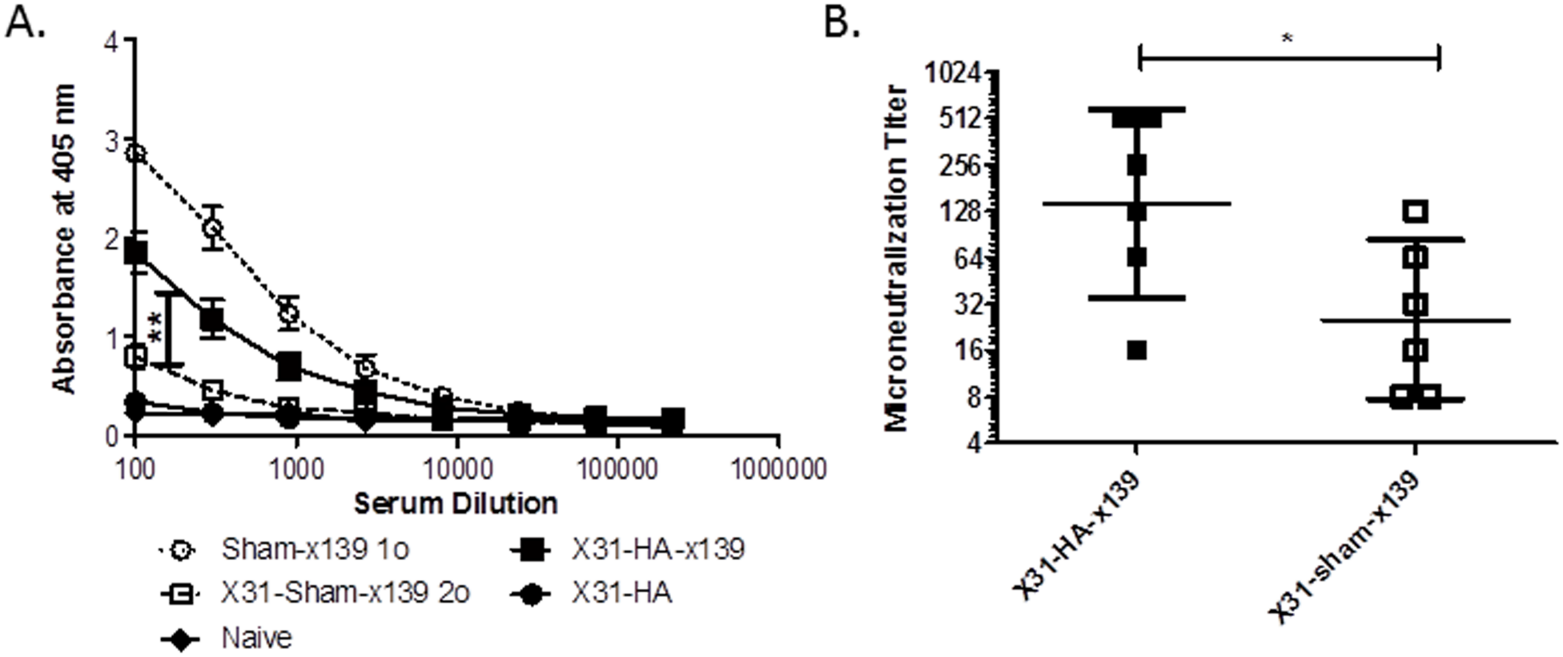
Establishing H1-specific CD4 T cell memory prior to secondary challenge partially restores the H1-specific antibody response. **A:** Mice were infected and immunized as depicted in Fig 3 and serum antibody responses were measured by H1 HA ELISA assay at day 8 following secondary infection. Data demonstrate the average OD from serum obtained from between 8–10 individual mice in the X31-HA, X31-HA-x139, and X31-sham-x139 groups and 3 individual mice in the naïve and sham-x139 groups, with error bars depicting the standard error of the mean. ** = p<0.01 using a linear mixed effect model for pairwise group comparisons. **B:** Mice were infected and immunized as depicted in Fig 3 and serum neutralizing antibody responses were determined by microneutralization assay at day 24 following secondary infection. Squares represent the neutralizing antibody titer in individual mice, with the geometric mean of the microneutralization titer shown by a line and error bars depicting the 95% confidence interval. * = p<0.05 by Wilcoxon rank-sum test.

We then sought to determine whether the presence of preexisting memory CD4 T cells against the HA protein led to an increase in HA-specific antibody following secondary infection. To examine this, ELISA assays were employed to measure the amount of HA-specific antibody present at day 8 following x139 infection. As shown in Fig 5A and in agreement with previous studies, there was a decrease in antibody against the H1 protein following a secondary infection in sham immunized mice (open squares) compared to the antibody titer detectable after primary infection (Group 4 (sham-x139) in Fig 3, open circles, p<0.0001). Importantly, prior immunization with a pool of HA peptides led to a statistically significant restoration of this H1-specific antibody response (black squares) when compared to the sham immunized group undergoing secondary infection (open squares; p = 0.009), although the titers achieved were not fully restored to the level seen following a primary infection (open circles, p = 0.07). When the serum dilution of antibody needed to produce an OD_405_ of 0.8 was quantified, we found that 9 times as much antibody was required to reach this OD in sham compared to HA preprimed mice. This increase in HA-specific antibodies occurred despite no evidence that an H1-specific B cell response was initiated by either the X-31 infection or the H1 peptide immunization (black circles). These results support our conclusion that establishing a memory CD4 T cell response against the HA protein prior to secondary challenge was sufficient to restore B cell reactivity to novel epitopes and enhance HA-specific antibody production upon heterosubtypic infection.

Finally, microneutralization assays were utilized to determine whether this HA prepriming strategy also led to an increase in neutralizing antibody. We found that the neutralizing antibody titer increased about 5.6 fold in mice with previously established HA-specific CD4 T cell memory when compared to sham-immunized mice that underwent a secondary influenza infection, from a geometric mean of 25.4 to a geometric mean of 143.7 (Fig 5B). This increase was of statistical significance (p = 0.036) in a pairwise comparison. No neutralizing antibody was detected in mice initially infected with X-31 and then immunized with the pool of H1 HA peptides (data not shown), indicating that neither cross reactive neutralizing antibody produced following infection with the highly divergent H3 virus nor B cell priming during the peptide immunization were responsible for this increased neutralizing antibody production.

To evaluate whether boosting of the response to the original H3 virus occurred at the expense of H1 antibody production on either immunization with a pool of H1 HA peptides or on secondary infection with x139 (original antigenic sin), antibody titers against the A/Brisbane/10/07 H3 HA protein were determined using an ELISA assay. While this HA protein was not identical to the HA protein contained within the X-31 virus, it was of the same family, allowing us to detect an increase in H3 antibody following challenge with the H1 virus. Antibody levels against the H3 protein were statistically indistinguishable in all groups infected with X-31 regardless of their exposure to H1 peptides or infection with the x139 virus. This suggests that H3 antibody titers were not boosted by either the H1 peptide immunization or infection with the x139 virus.

## Discussion

The studies reported here demonstrate that the decrease in both HA-specific CD4 T cells and antibody following a secondary heterosubtypic infection can be partially restored solely by establishing memory CD4 T cells against the novel HA protein prior to secondary infection. These HA-specific CD4 T cells are likely to provide enhanced help to B cells specific for the HA protein, leading to increases in both total HA-specific as well as neutralizing antibodies. This finding provides an argument for vaccination strategies that promote broadly cross reactive HA-specific CD4 T cell immunity, which could enable robust antibody production in response to viruses or vaccines composed of novel and potentially pandemic influenza strains against which the host has few preexisting neutralizing antibodies.

One possible mechanism underlying the failure to prime naïve CD4 T cells in this mouse model is the rapid clearance of antigen that occurs following this heterosubtypic infection. It is known that memory CD4 T cells are able to rapidly recruit innate immune effectors to the lung following infection, leading to enhanced viral clearance and decreasing the abundance of antigen [24–26]. Lower antigenic load will have a disproportionate effect on naïve CD4 T cells [32–34], likely leading to diminished recruitment and expansion of this population. As antigenic drift mostly occurs in the surface glycoproteins, this will have the greatest impact on HA- and NA-specific CD4 T cells. As both of these specificities and their associated antibody response play important roles in the protecting from infection, these alterations could be detrimental to the development of immunologic protection, especially against more novel viral strains. Another potential factor contributing to the decreased immunological reactivity to HA following secondary infection could be a diminished inflammatory response following secondary infection. This could be interesting to explore in future studies.

While we were able to restore HA-specific antibody by establishing CD4 T cell memory to the HA protein, the restoration of the antibody response was only partial. Although we established a broad repertoire of anti-HA CD4 T cell reactivity, it is important to note that only 5 of the 13 major HA peptide-epitopes were included in our peptide vaccination regimen. Further, although robust numbers of memory cells were established in the spleen post-vaccination, it is likely that only a subset of these cells were recruited into the draining lymph node upon heterosubtypic challenge. We hypothesize that this partial restoration of the antibody response was enabled by an increase in HA-specific Tfh in HA immunized mice. This hypothesis is supported by previous studies by our laboratory demonstrating that an antecedent peptide immunization was able to increase Tfh and germinal center B cells at day 7 post infection relative to that generated by a primary infection. These enhanced Tfh cells were associated with an antigen specific increase in antibody. Further, there was a correlation between Tfh numbers and the number of germinal center B cells, supporting a limiting role for Tfh in the germinal center reaction [39]. Additional studies to quantify CD4 T cell specificity within the Tfh pool in this secondary challenge model are currently underway.

**Fig 6:**
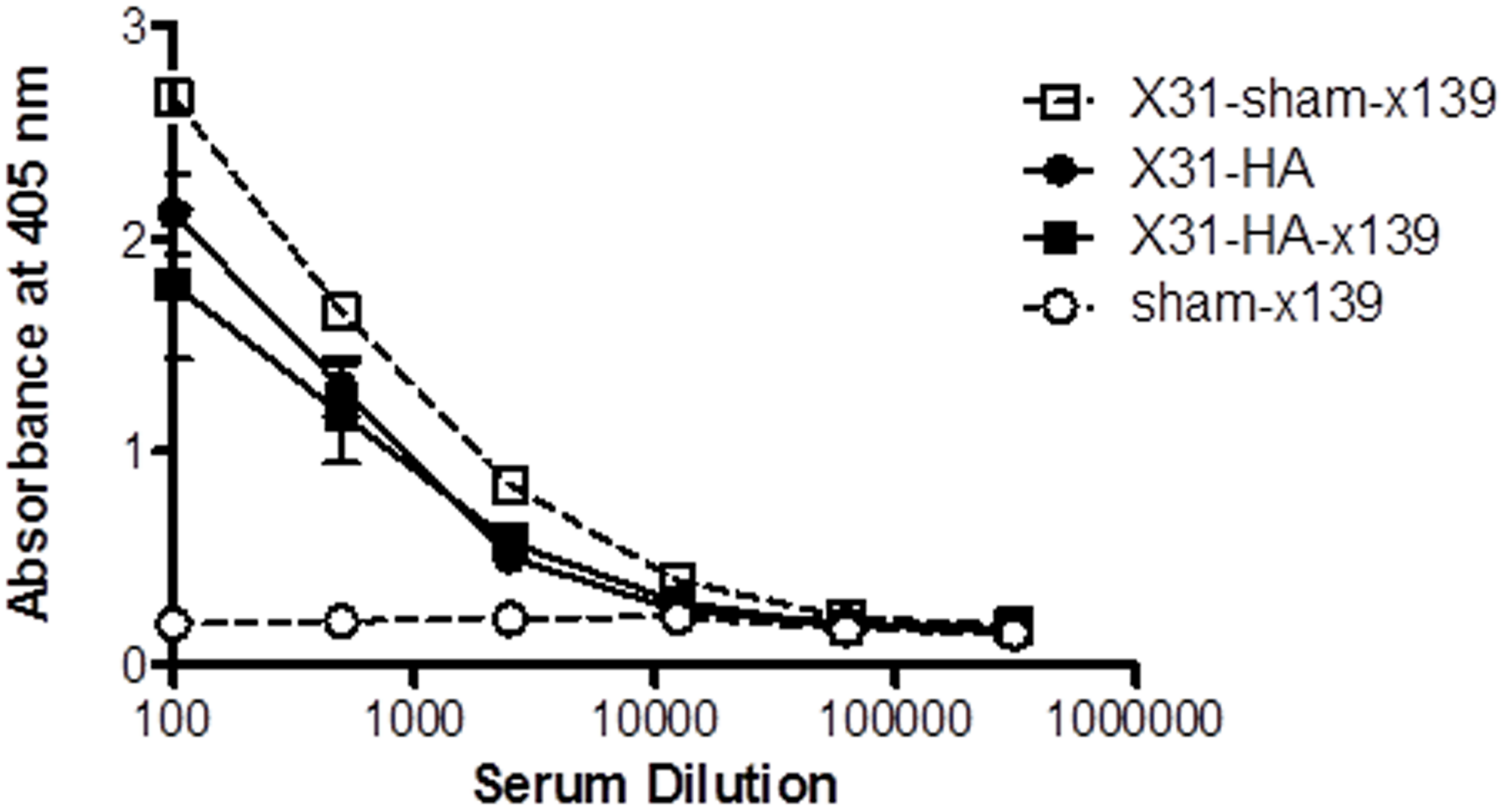
H3-specific antibody responses are not boosted following secondary infection with the x139 H1N1 virus. To evaluate for evidence of original antigenic sin, serum from mice in each group was tested for antibody against the A/Brisbane/10/07 H3 HA protein by ELISA assay at 24 days following x139 viral challenge. Data represent the average OD obtained at a given serum dilution from 5–6 individual mice per experimental group, with the exception of the sham-x139 group where only 2 mice were examined. There were no statistically significant differences between H3 antibody levels in the sham-immunized (X31-sham-x139) or HA-immunized (X31-HA-x139) mice when compared to antibody in the mice initially primed with the X31 virus and immunized with HA peptides without a subsequent H1 viral infection (X31-HA).

It is interesting to consider our results with regard to the phenomenon of original antigenic sin as has been described for influenza virus. Traditionally, original antigenic sin following a secondary influenza challenge occurs when the host is exposed to a second, closely related influenza strain, resulting in a preferential antibody response to epitopes expressed within the initial viral strain [45–48]. It is now generally accepted that in both B and T cell immunity, memory cells have a considerable advantage both in terms of abundance and the threshold of activation needed to induce a response. Indeed, original antigenic sin has been described in CD8 T cells following infections with related strains of LCMV [49] and Dengue virus [50]. As highly divergent HA proteins from different groups (“X-31” H3; “x139” H1) were used in our challenge model, infection with the H3 viral strain did not induce detectable levels of cross reactive H1 antibody by either ELISA (Fig 5A) or microneutralization assay, and no boosting of H3 antibody was detectable on H1 challenge (Fig 6), our findings do not fit the traditional definition of original antigenic sin. However, these findings highlight the need to think more broadly about how prior exposure influences all aspects of immunity, possibly leading to a broader definition of how original antigenic sin shapes the adaptive immune response following repeated challenge with influenza virus.

One of the advantages of this study is the use of peptide-based priming with known CD4 T cell epitopes. This provided monovalent, unstructured ligands with poor B cell-stimulatory capacity that were able to expand the CD4 T cell repertoire without the generation of B cell memory or the establishment of HA-specific antibody that might neutralize the secondary virus [39]. That such focused priming of HA-specific CD4 T cells was able to partially restore B cell reactivity to HA provides additional evidence supporting a linkage between CD4 T cell and B cell specificities [14,39,51,52]. Such a linkage likely occurs by selective internalization of only the viral proteins engaged by the immunoglobulin receptor and subsequent presentation of a limited diversity of CD4 T cell epitopes. In this situation, HA-specific B cells will predominantly display epitopes derived from the HA protein, and thus will only be able to recruit help from HA-specific CD4 T cells.

Although almost all of the human population has immunologic memory that cross reacts with seasonal influenza viruses, conserved HA epitopes may be more limited between divergent influenza viruses. Our study demonstrates that priming HA-specific memory cells prior to secondary challenge establishes a population of cells able to be recalled and provide help to HA-specific B cells even in the setting of a low antigenic load. As CD4 T cell epitopes are substantially more conserved among serologically distinct HA proteins, the ability of preexisting HA-specific memory CD4 T cells to increase the anti-HA antibody response supports the use of vaccination strategies that establish a diverse memory CD4 T cell repertoire that includes cells reactive against potentially pandemic HA proteins. This preexisting pool of HA-specific memory cells could then be recruited on exposure to even a highly divergent virus against which there is little preexisting B cell memory, enhancing antibody production during an active infection and possibly allowing for neutralizing antibodies to be generated more rapidly upon immunization with a pandemic vaccine. Such selective arming of the CD4 T cell compartment thus has the potential to decrease infection rates, morbidity, and mortality on the emergence of the next influenza pandemic.

## References

[1] Thompson WW, Shay DK, Weintraub E, Brammer L, Bridges CB, Cox NJ, et al (2004) Influenza-associated hospitalizations in the United States. JAMA 292: 1333–1340. 10.1001/jama.292.11.1333 15367555

[2] Thompson WW, Shay DK, Weintraub E, Brammer L, Cox N, Anderson LJ, et al (2003) Mortality associated with influenza and respiratory syncytial virus in the United States. JAMA 289: 179–186. 12517228 10.1001/jama.289.2.179

[3] Nguyen JL, Yang W, Ito K, Matte TD, Shaman J, Kinney PL (2016) Seasonal Influenza Infections and Cardiovascular Disease Mortality. JAMA Cardiol 1: 274–281. 10.1001/jamacardio.2016.0433 27438105 PMC5158013

[4] Heikkinen T, Silvennoinen H, Peltola V, Ziegler T, Vainionpaa R, Vuorinen T, et al (2004) Burden of influenza in children in the community. J Infect Dis 190: 1369–1373. 10.1086/424527 15378427

[5] Wong KK, Cheng P, Foppa I, Jain S, Fry AM, Finelli L,. (2015) Estimated paediatric mortality associated with influenza virus infections, United States, 2003–2010. Epidemiol Infect 143: 640–647. 10.1017/S0950268814001198 24831613 PMC9507064

[6] Jain S, Self WH, Wunderink RG, Team CES (2015) Community-Acquired Pneumonia Requiring Hospitalization. N Engl J Med 373: 2382 10.1056/NEJMc1511751 26650159 PMC9338768

[7] Couch RB (2008) Seasonal inactivated influenza virus vaccines. Vaccine 26 Suppl 4: D5–9. 18602728 10.1016/j.vaccine.2008.05.076 PMC2643340

[8] Peiris JS, Poon LL, Guan Y (2009) Emergence of a novel swine-origin influenza A virus (S-OIV) H1N1 virus in humans. J Clin Virol 45: 169–173. 10.1016/j.jcv.2009.06.006 19540800 PMC4894826

[9] Dawood FS, Jain S, Finelli L, Shaw MW, Lindstrom S, Garten RJ, et al (2009) Emergence of a novel swine-origin influenza A (H1N1) virus in humans. New England Journal of Medicine 360: 2605–2615. 10.1056/NEJMoa0903810 19423869

[10] Richards KA, Chaves FA, Krafcik FR, Topham DJ, Lazarski CA, Sant AJ,. (2007) Direct ex vivo analyses of HLA-DR1 transgenic mice reveal an exceptionally broad pattern of immunodominance in the primary HLA-DR1-restricted CD4 T-cell response to influenza virus hemagglutinin. Journal of virology 81: 7608–7619. 10.1128/JVI.02834-06 17507491 PMC1933370

[11] Richards KA, Chaves FA, Sant AJ (2009) Infection of HLA-DR1 transgenic mice with a human isolate of influenza a virus (H1N1) primes a diverse CD4 T-cell repertoire that includes CD4 T cells with heterosubtypic cross-reactivity to avian (H5N1) influenza virus. J Virol 83: 6566–6577. 10.1128/JVI.00302-09 19386707 PMC2698557

[12] Nayak JL, Richards KA, Chaves FA, Sant AJ (2010) Analyses of the specificity of CD4 T cells during the primary immune response to influenza virus reveals dramatic MHC-linked asymmetries in reactivity to individual viral proteins. Viral Immunology 23: 169–180. 10.1089/vim.2009.0099 20373997 PMC2883523

[13] Crowe SR, Miller SC, Brown DM, Adams PS, Dutton RW, Harmsen AG, et al (2006) Uneven distribution of MHC class II epitopes within the influenza virus. Vaccine 24: 457–467. 10.1016/j.vaccine.2005.07.096 16140434

[14] Nayak JL, Alam S, Sant AJ (2013) Cutting edge: Heterosubtypic influenza infection antagonizes elicitation of immunological reactivity to hemagglutinin. Journal of Immunology 191: 1001–1005. 10.4049/jimmunol.1203520 PMC6728918 23794632

[15] Nayak JL, Fitzgerald TF, Richards KA, Yang H, Treanor JJ Sant AJ,. (2013) CD4+ T-cell expansion predicts neutralizing antibody responses to monovalent, inactivated 2009 pandemic influenza A(H1N1) virus subtype H1N1 vaccine. J Infect Dis 207: 297–305. 10.1093/infdis/jis684 23148285 PMC3532833

[16] MacLeod MK, Kappler JW, Marrack P (2010) Memory CD4 T cells: generation, reactivation and re-assignment. Immunology 130: 10–15. 10.1111/j.1365-2567.2010.03260.x 20331469 PMC2855788

[17] McKinstry KK, Strutt TM, Swain SL (2010) The potential of CD4 T-cell memory. Immunology 130: 1–9. 10.1111/j.1365-2567.2010.03259.x 20331470 PMC2855787

[18] Croft M, Bradley LM, Swain SL (1994) Naive versus memory CD4 T cell response to antigen. Memory cells are less dependent on accessory cell costimulation and can respond to many antigen-presenting cell types including resting B cells. Journal of Immunology 152: 2675–2685. 7908301

[19] Rogers PR, Dubey C, Swain SL (2000) Qualitative changes accompany memory T cell generation: faster, more effective responses at lower doses of antigen. Journal of Immunology 164: 2338–2346. 10.4049/jimmunol.164.5.2338 10679068

[20] von Essen MR, Kongsbak M, Geisler C (2012) Mechanisms behind functional avidity maturation in T cells. Clin Dev Immunol 2012: 163453 10.1155/2012/163453 22611418 PMC3351025

[21] Brown DM, Dilzer AM, Meents DL, Swain SL (2006) CD4 T cell-mediated protection from lethal influenza: perforin and antibody-mediated mechanisms give a one-two punch. The journal of immunology 177: 2888–2898. 16920924 10.4049/jimmunol.177.5.2888

[22] Swain SL, Agrewala JN, Brown DM, Jelley-Gibbs DM, Golech S, Huston G, et al (2006) CD4+ T-cell memory: generation and multi-faceted roles for CD4+ T cells in protective immunity to influenza. Immunol Rev 211: 8–22. 10.1111/j.0105-2896.2006.00388.x 16824113 PMC2266984

[23] Belz GT, Wodarz D, Diaz G, Nowak MA, Doherty PC (2002) Compromised influenza virus-specific CD8(+)-T-cell memory in CD4(+)-T-cell-deficient mice. Journal of Virology 76: 12388–12393. 10.1128/JVI.76.23.12388-12393.2002 12414983 PMC136883

[24] Teijaro JR, Turner D, Pham Q, Wherry EJ, Lefrancois L, Farber DL (2011) Cutting edge: Tissue-retentive lung memory CD4 T cells mediate optimal protection to respiratory virus infection. Journal of Immunology 187: 5510–5514. 10.4049/jimmunol.1102243 PMC3221837 22058417

[25] Teijaro JR, Verhoeven D, Page CA, Turner D, Farber DL (2010) Memory CD4 T cells direct protective responses to influenza virus in the lungs through helper-independent mechanisms. Journal of Virology 84: 9217–9226. 10.1128/JVI.01069-10 20592069 PMC2937635

[26] Strutt TM, McKinstry KK, Dibble JP, Winchell C, Kuang Y, Curtis JD (2010) Memory CD4+ T cells induce innate responses independently of pathogen. Nature Medicine 16: 558–564, 551 p following 564. 10.1038/nm.2142 20436484 PMC2927232

[27] Strutt TM, McKinstry KK, Marshall NB, Vong AM, Dutton RW, Swain SL (2013) Multipronged CD4(+) T-cell effector and memory responses cooperate to provide potent immunity against respiratory virus. Immunol Rev 255: 149–164. 10.1111/imr.12088 23947353 PMC4206082

[28] McKinstry KK, Dutton RW, Swain SL, Strutt TM (2013) Memory CD4 T cell-mediated immunity against influenza A virus: more than a little helpful. Arch Immunol Ther Exp (Warsz) 61: 341–353. 23708562 10.1007/s00005-013-0236-z PMC3874125

[29] Yang J, Huck SP, McHugh RS, Hermans IF, Ronchese F (2006) Perforin-dependent elimination of dendritic cells regulates the expansion of antigen-specific CD8+ T cells in vivo. Proceedings of the National Academy of Sciences of the United States of America 103: 147–152. 10.1073/pnas.0509054103 16373503 PMC1324995

[30] Belz GT, Zhang L, Lay MD, Kupresanin F, Davenport MP (2007) Killer T cells regulate antigen presentation for early expansion of memory, but not naive, CD8+ T cell. Proceedings of the National Academy of Sciences of the United States of America 104: 6341–6346. 10.1073/pnas.0609990104 17400753 PMC1840050

[31] Ravkov EV, Williams MA (2009) The magnitude of CD4+ T cell recall responses is controlled by the duration of the secondary stimulus. Journal of Immunology 183: 2382–2389. 10.4049/jimmunol.0900319 PMC2819348 19605694

[32] Obst R, van Santen HM, Mathis D, Benoist C (2005) Antigen persistence is required throughout the expansion phase of a CD4(+) T cell response. Journal of Experimental Medicine 201: 1555–1565. 10.1084/jem.20042521 15897273 PMC2212918

[33] Obst R, van Santen HM, Melamed R, Kamphorst AO, Benoist C, Matthis D (2007) Sustained antigen presentation can promote an immunogenic T cell response, like dendritic cell activation. Proceedings of the National Academy of Sciences of the United States of America 104: 15460–15465. 10.1073/pnas.0707331104 17881563 PMC2000557

[34] Yarke CA, Dalheimer SL, Zhang N, Catron DM, Jenkins MK, Mueller DL (2008) Proliferating CD4+ T cells undergo immediate growth arrest upon cessation of TCR signaling in vivo. J Immunol 180: 156–162. 18097015 10.4049/jimmunol.180.1.156

[35] Suh WK (2015) Life of T follicular helper cells. Mol Cells 38: 195–201. 10.14348/molcells.2015.2331 25537859 PMC4363718

[36] Ueno H, Banchereau J, Vinuesa CG (2015) Pathophysiology of T follicular helper cells in humans and mice. Nat Immunol 16: 142–152. 10.1038/ni.3054 25594465 PMC4459756

[37] McHeyzer-Williams LJ, Pelletier N, Mark L, Fazilleau N, McHeyzer-Williams MG (2009) Follicular helper T cells as cognate regulators of B cell immunity. Current Opinion in Immunology 21: 266–273. 10.1016/j.coi.2009.05.010 19502021 PMC2731669

[38] Ramiscal RR, Vinuesa CG (2013) T-cell subsets in the germinal center. Immunol Rev 252: 146–155. 10.1111/imr.12031 23405902

[39] Alam S, Knowlden ZA, Sangster MY, Sant AJ (2014) CD4 T cell help is limiting and selective during the primary B cell response to influenza virus infection. J Virol 88: 314–324. 10.1128/JVI.02077-13 24155379 PMC3911719

[40] Bentebibel SE, Lopez S, Obermoser G, Schmitt N, Mueller C, Harrod C, et al (2013) Induction of ICOS+CXCR3+CXCR5+ TH cells correlates with antibody responses to influenza vaccination. Sci Transl Med 5: 176ra132. 10.1126/scitranslmed.3005191 PMC3621097 23486778

[41] Herati RS, Reuter MA, Dolfi DV, Mansfield KD, Aung H, Badwan OZ, et al (2014) Circulating CXCR5+PD-1+ response predicts influenza vaccine antibody responses in young adults but not elderly adults. J Immunol 193: 3528–3537. 10.4049/jimmunol.1302503 25172499 PMC4170011

[42] Crotty S (2011) Follicular helper CD4 T cells (TFH). Annu Rev Immunol 29: 621–663. 10.1146/annurev-immunol-031210-101400 21314428

[43] Treanor J, Wright PF (2003) Immune correlates of protection against influenza in the human challenge model. Dev Biol (Basel) 115: 97–104. 15088780

[44] Alam S, Sant AJ (2011) Infection with seasonal influenza virus elicits CD4 T cells specific for genetically conserved epitopes that can be rapidly mobilized for protective immunity to pandemic H1N1 influenza virus. Journal of Virology 85: 13310–13321. 10.1128/JVI.05728-11 21976658 PMC3233140

[45] Cobey S, Hensley SE (2017) Immune history and influenza virus susceptibility. Curr Opin Virol 22: 105–111. 10.1016/j.coviro.2016.12.004 28088686 PMC5467731

[46] Kim JH, Skountzou I, Compans R, Jacob J (2009) Original antigenic sin responses to influenza viruses. Journal of Immunology 183: 3294–3301. 10.4049/jimmunol.0900398 PMC4460008 19648276

[47] Fazekas de St G, Webster RG (1966) Disquisitions of Original Antigenic Sin. I. Evidence in man. Journal of Experimental Medicine 124: 331–345. 5922742 10.1084/jem.124.3.331 PMC2138235

[48] Fonville JM, Wilks SH, James SL, Fox A, Ventresca M, Aban M, et al (2014) Antibody landscapes after influenza virus infection or vaccination. Science 346: 996–1000. 10.1126/science.1256427 25414313 PMC4246172

[49] Klenerman P, Zinkernagel RM (1998) Original antigenic sin impairs cytotoxic T lymphocyte responses to viruses bearing variant epitopes. Nature 394: 482–485. 10.1038/28860 9697771

[50] Mongkolsapaya J, Dejnirattisai W, Xu XN, Vasanawathana S, Tangthawornchaikul N, Chairunsri A, et al (2003) Original antigenic sin and apoptosis in the pathogenesis of dengue hemorrhagic fever. Nat Med 9: 921–927. 10.1038/nm887 12808447

[51] Sette A, Moutaftsi M, Moyron-Quiroz J, McCausland MM, Davies DH, Johnston RJ, et al (2008) Selective CD4+ T cell help for antibody responses to a large viral pathogen: deterministic linkage of specificities. Immunity 28: 847–858. 10.1016/j.immuni.2008.04.018 18549802 PMC2504733

[52] Nayak JL, Richards KA, Yang H, Treanor JJ, Sant AJ (2014) Effect of Influenza A(H5N1) Vaccine Prepandemic Priming on CD4+ T-Cell Responses. J Infect Dis. 10.1093/infdis/jiu616 PMC4425838 25378637

